# Letter from London — Esmarch’s Method of Controlling Hemorrhage

**Published:** 1873-11

**Authors:** 


*WESTERN LANCET.*

Letter from G. L. Simmons, M.D., of Sacramento, from London, England.—11 *Queen Square, Bloomsbury, W. C., London, October* 12*th*, 1873.—Dear Doctor: I will fulfill my promise to write to you from abroad, in case I saw anything of interest by calling the attention of your surgical readers to Esmarch's method of suppressing hemorrhage during operations upon the extremities, as easily and minutely upon the living as upon the dead subject. As you are doubtless aware, the method consists in the simple application (from the toes or fingers upwards) of an elastic bandage two and one-half inches in width. As the turns of the bandage ascend, by the exercise of considerable force the blood is driven from the veins and arteries, and when any desired point is reached, a piece of half inch rubber tubing is brought two or three times tightly around the limb just above the bandage, and tied. The bandage is then removed.

By the kindness of Mr. T. Smith, who now fills the post of surgeon at St. Bartholomew's Hospital, made vacant by the death of Holmes Coote. I was invited to see him operate in a case where the circulation was suppressed by the elastic bandage. A boy with a diseased tibia, was put under the influence of chloroform, the bandage applied, and the diseased bone removed. The experiment was so completely successful that no blood was lost during the operation, all of the tissues being blanched even to the osseous structure. I need hardly remind you, as remarked by Mr. Smith, "that this operation is usually made slow and troublesome, by the constant welling-up of blood.

Mr. Cripp, the house surgeon at St Bartholomew's, showed me a modification of Esmarch's method which discards the bandage, and relies upon a seven-inch ring of three-eighths inch rubber tubing for compression. After making a few turns at the very extremity of the limb, the tubing is carried upwards on a reel, and fastened where desired. A description of his modification is published in the *Lancet* of this date.

At King's College Hospital, I saw Mr. John Wood operate in a case of diseased elbow, where it was necessary to open long and numerous sinuses, and so exsanguined were the tissues, that not a drop of blood was evident, and the surgeon stated, that he could define the structures as clearly as if a dead limb were under his knife. He also mentioned a case of a late operation upon an arm, very near the axilla, where he had first forced all of the blood out of the limb by elastic pressure, and had then applied a pad over the axillary artery, keeping it in place by numerous turns of rubber tubing over the shoulder.

Although no bad effects have been evident from the adoption of this plan, in several of the leading hospitals in London, it has been suggested that so perfect and prolonged a pressure upon the vessels of a limb, might lead to the formation of a clot which being carried into the circulation, would lead to evil results. Others intimate the possibility of forcing septic matters into the veins, in certain cases.

To avoid this, in a case of amputation at the thigh, for gangrene of the foot, which occurred at St. Thomas' Hospital, the surgeon in charge, ordered the elastic bandaging to be commenced above the dead parts. Whatever may be thought of Esmarch's plan in amputations, there can be no doubt that it possesses special advantages in cases of operations upon tumors, sinuses or diseased bones of the extremities.

